# Supplementary figures and images for: T4 DNA polymerase prevents deleterious on-target DNA damage and enhances precise CRISPR editing
Source: EMBO J. 2024 Jul 22;43(17):3733–51. doi: 10.1038/s44318-024-00158-6 (PMC11377749; doi:10.1038/s44318-024-00158-6)

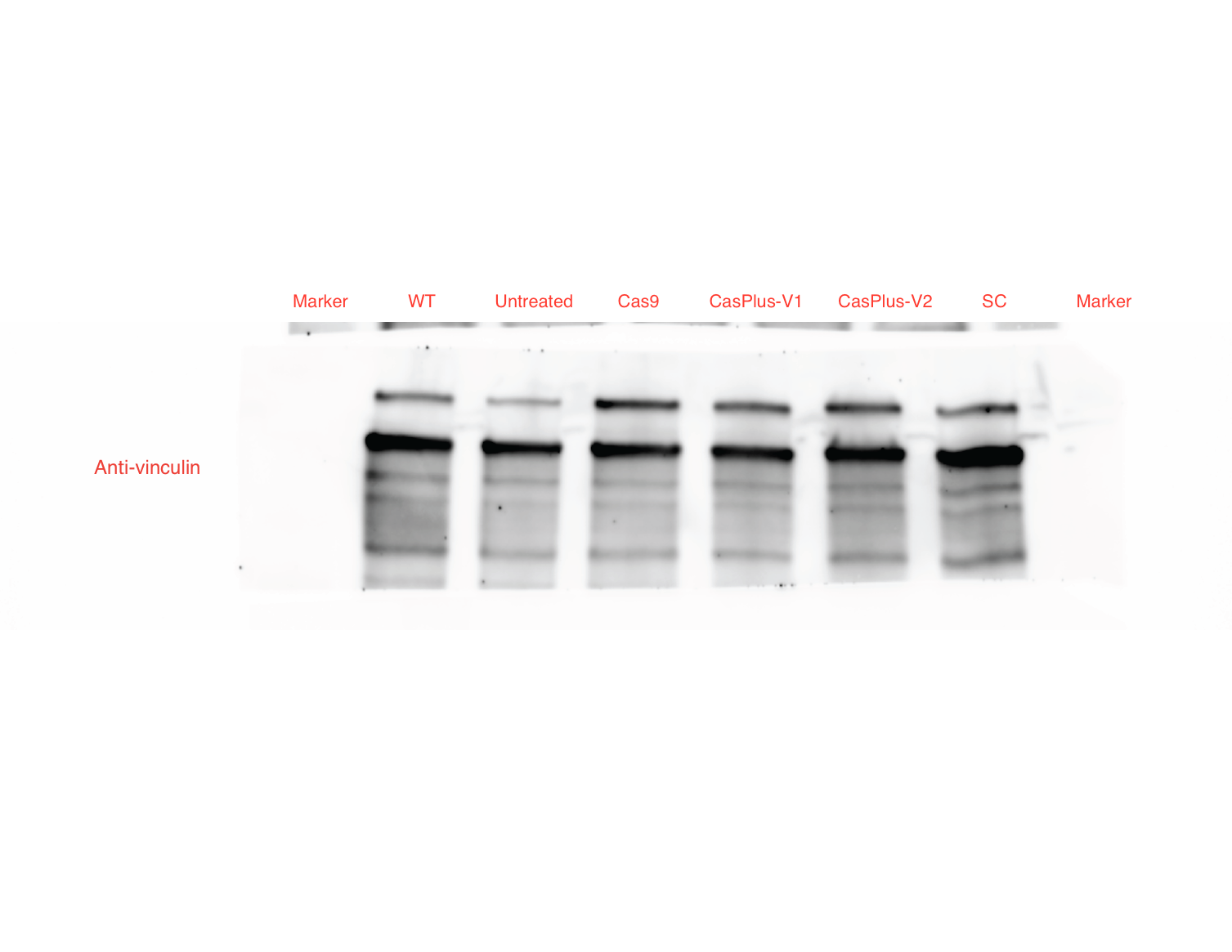

Supplement: Supplementary file 5 — Source data Fig. 3 [file 44318_2024_158_MOESM5_ESM.zip › SD figure 3/3F/Anti-vinculin-right panel.tiff]

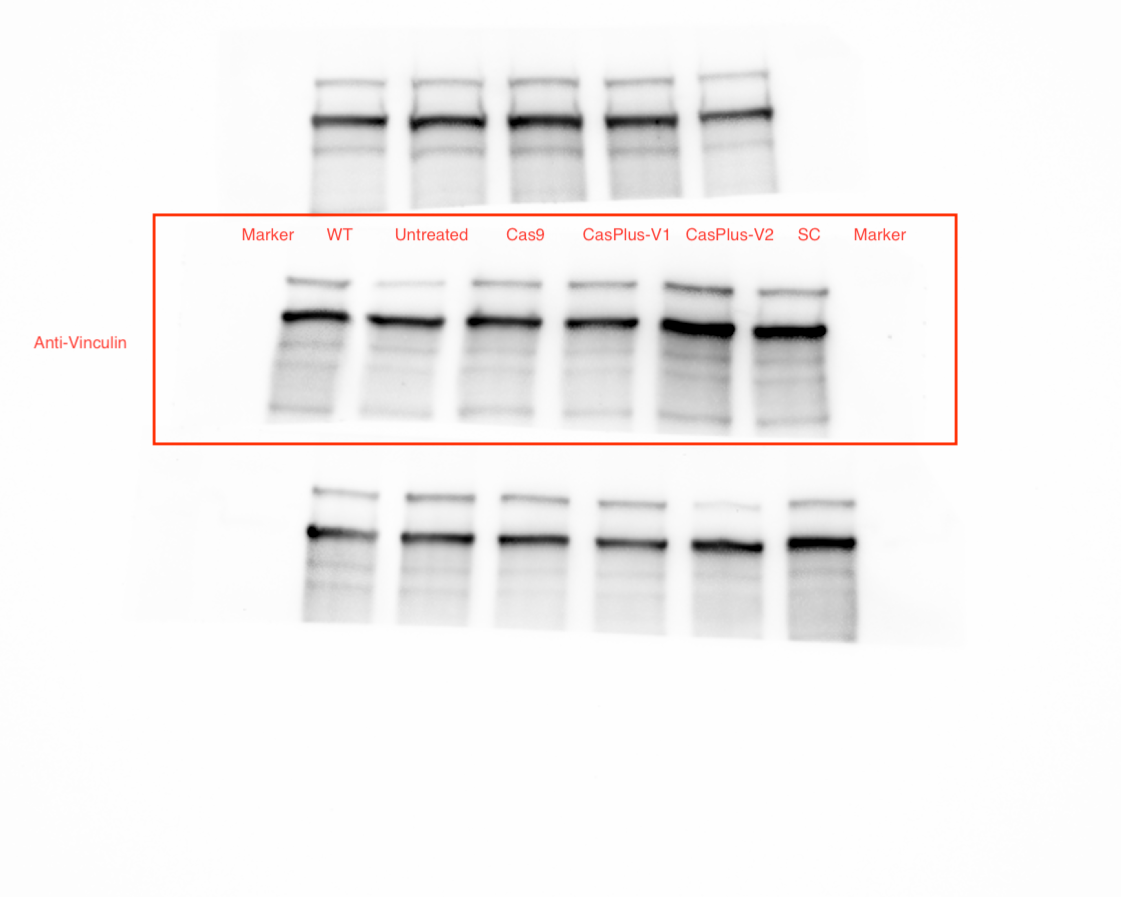

Supplement: Supplementary file 5 — Source data Fig. 3 [file 44318_2024_158_MOESM5_ESM.zip › SD figure 3/3F/Anti-Vinculin Left panel.tiff]

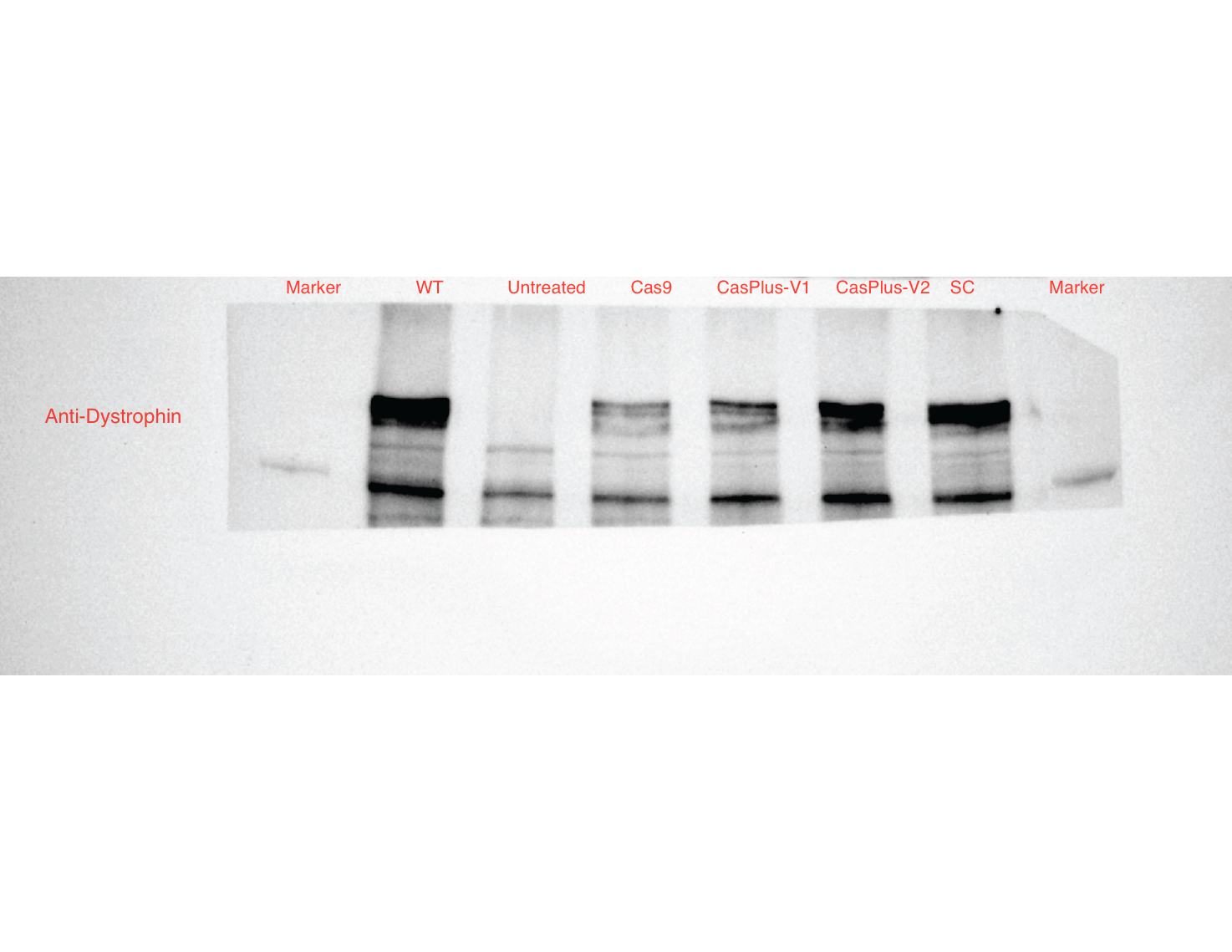

Supplement: Supplementary file 5 — Source data Fig. 3 [file 44318_2024_158_MOESM5_ESM.zip › SD figure 3/3F/Anti-Dystrophin-right panel.tiff]

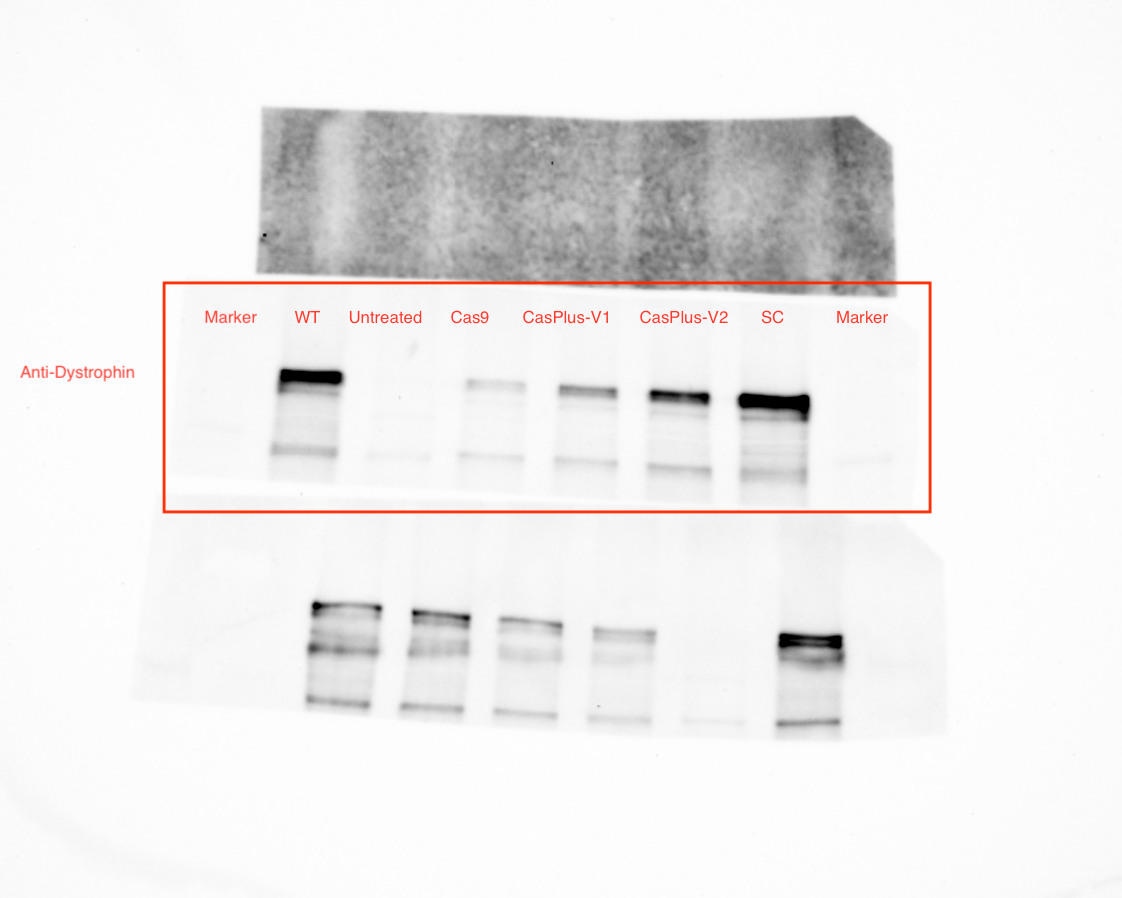

Supplement: Supplementary file 5 — Source data Fig. 3 [file 44318_2024_158_MOESM5_ESM.zip › SD figure 3/3F/Anti-Dystrophin Left panel.tiff]

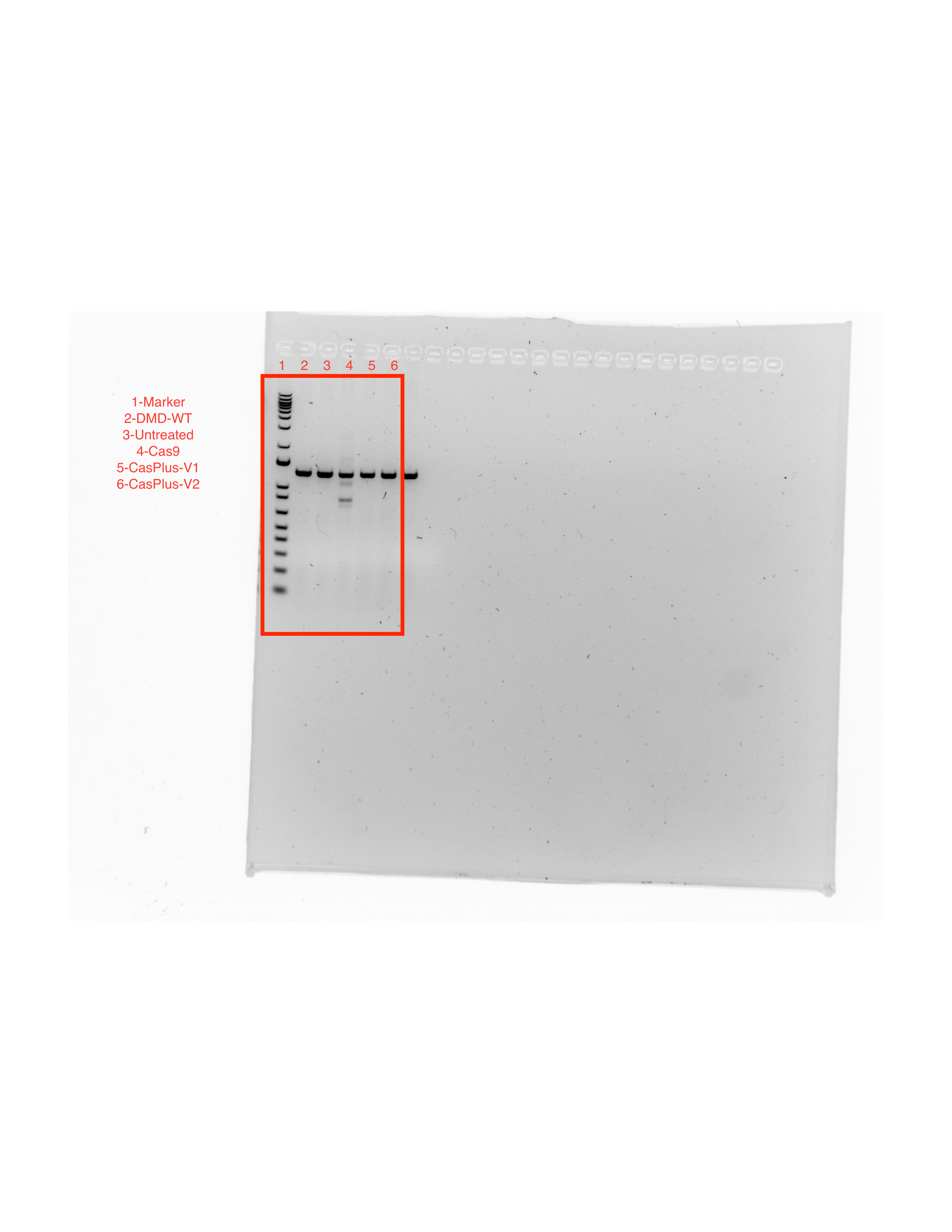

Supplement: Supplementary file 6 — Source data Fig. 4 [file 44318_2024_158_MOESM6_ESM.zip › SD figure 4/4B/4B-left.tiff]

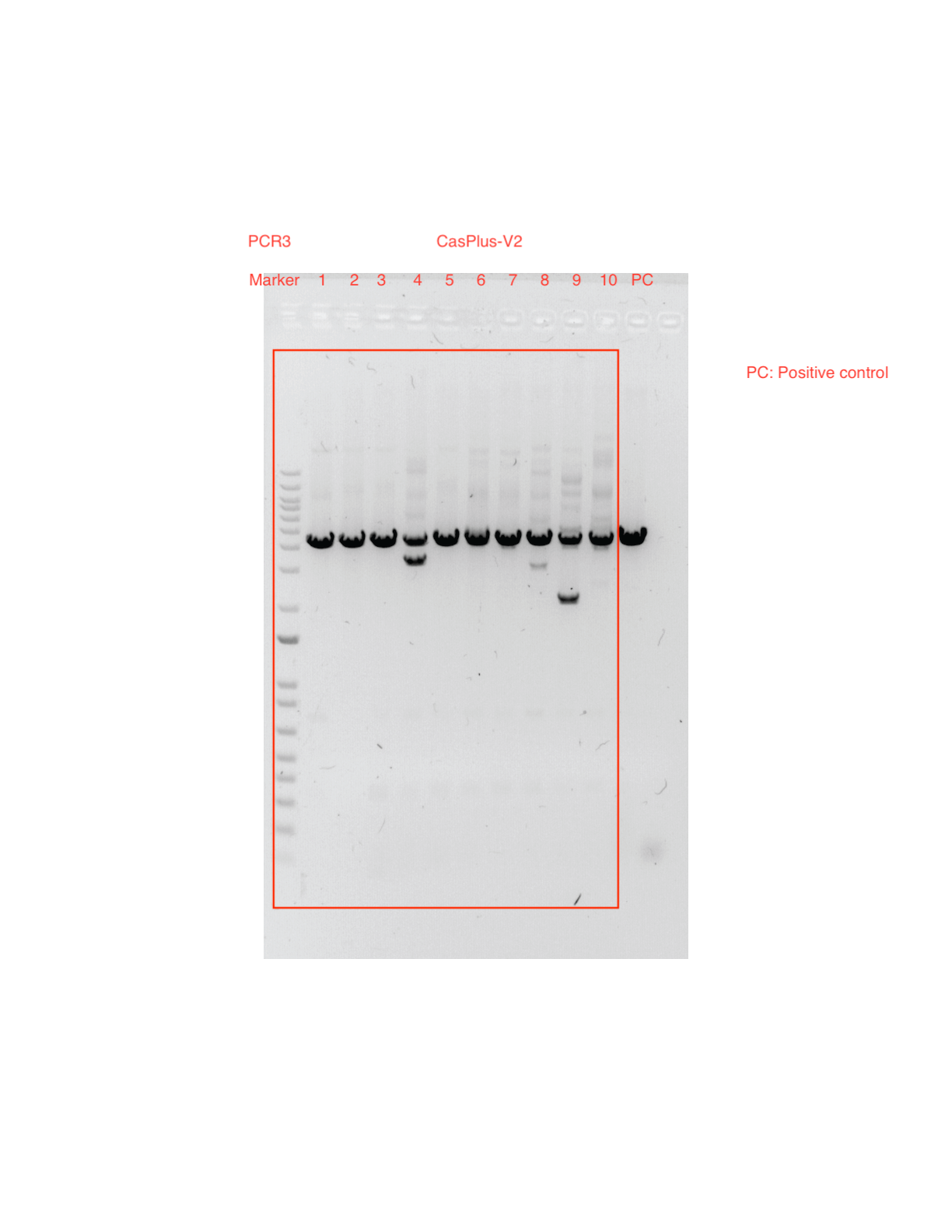

Supplement: Supplementary file 7 — Source data Fig. 5 [file 44318_2024_158_MOESM7_ESM.zip › SD figure 5/5B/5B-right.tiff]

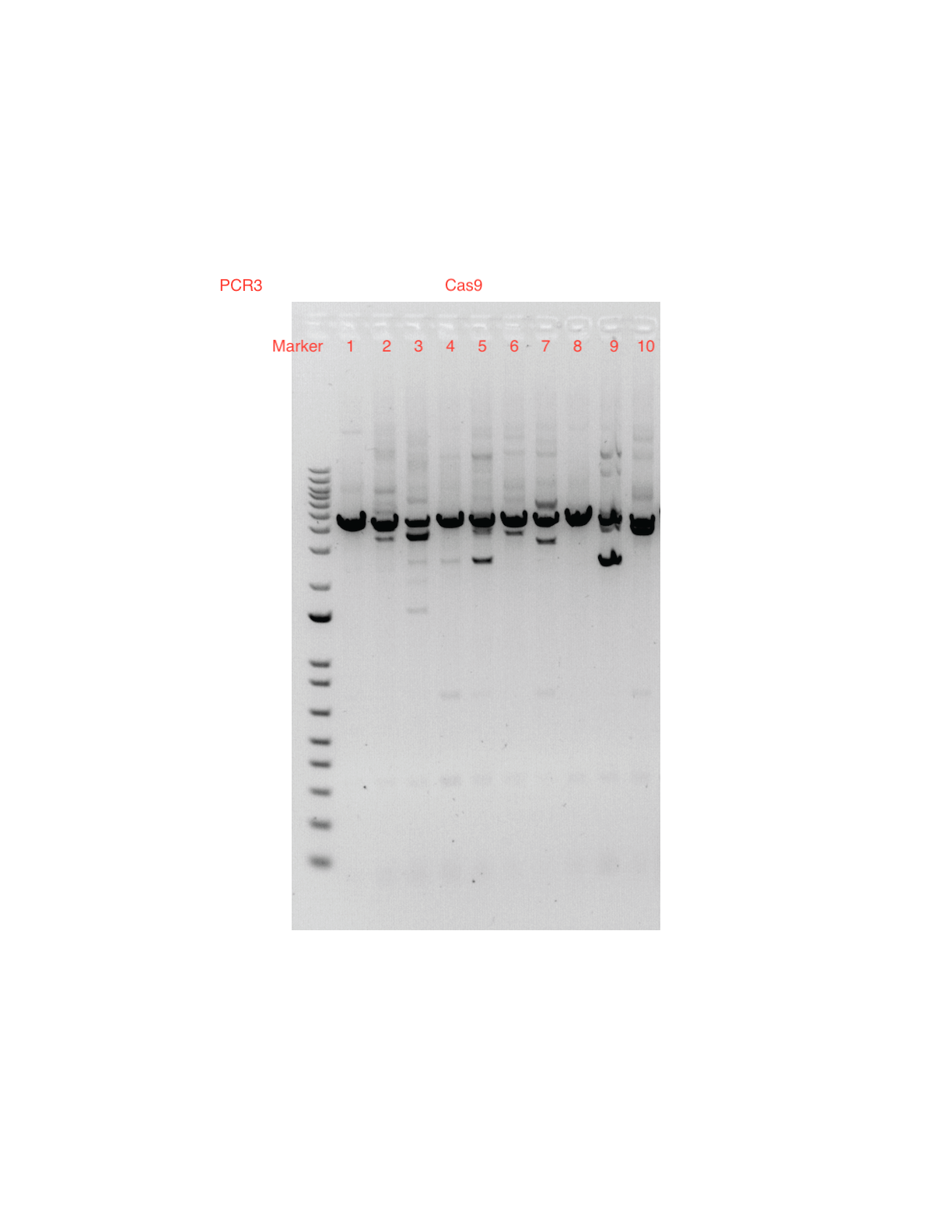

Supplement: Supplementary file 7 — Source data Fig. 5 [file 44318_2024_158_MOESM7_ESM.zip › SD figure 5/5B/5B-left.tiff]

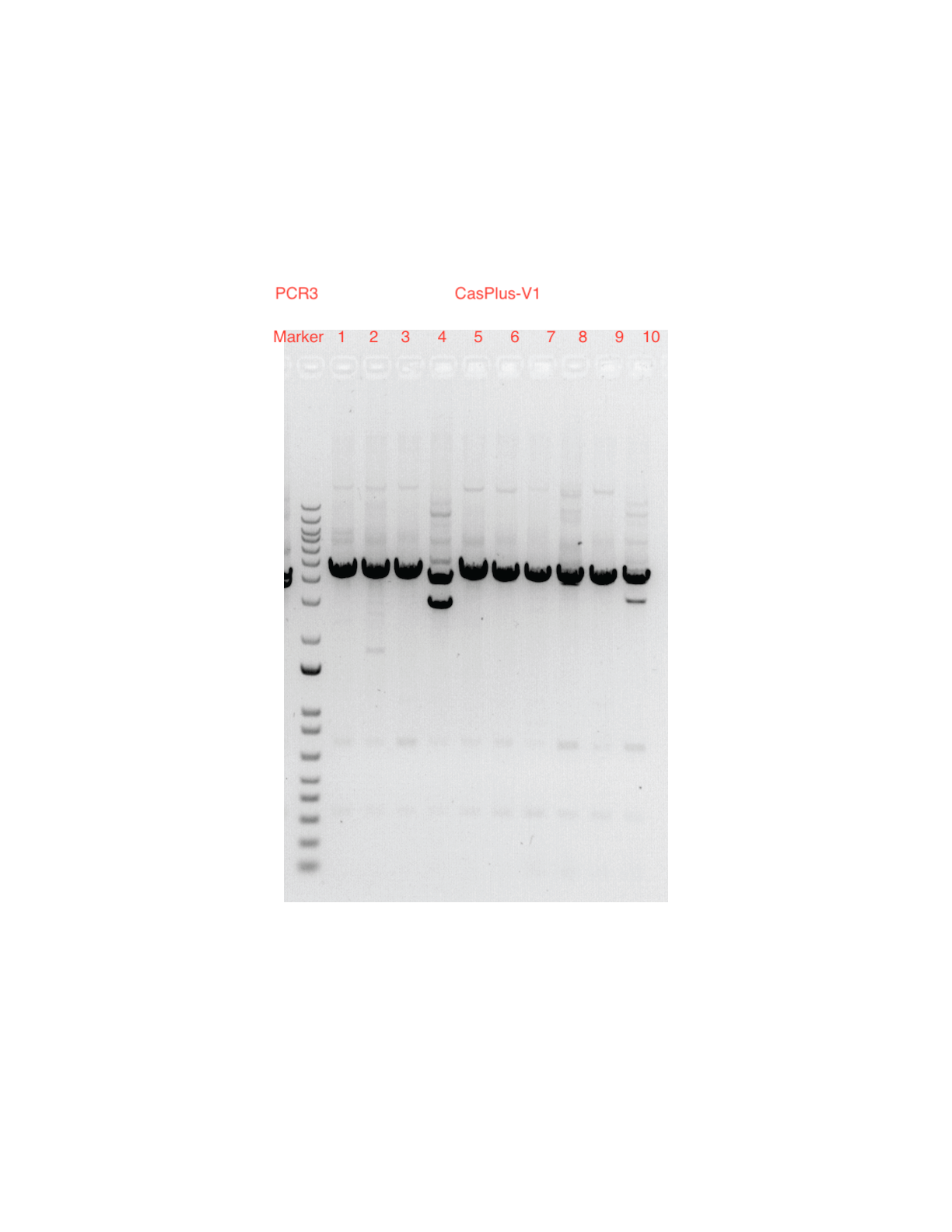

Supplement: Supplementary file 7 — Source data Fig. 5 [file 44318_2024_158_MOESM7_ESM.zip › SD figure 5/5B/5B-middle.tiff]

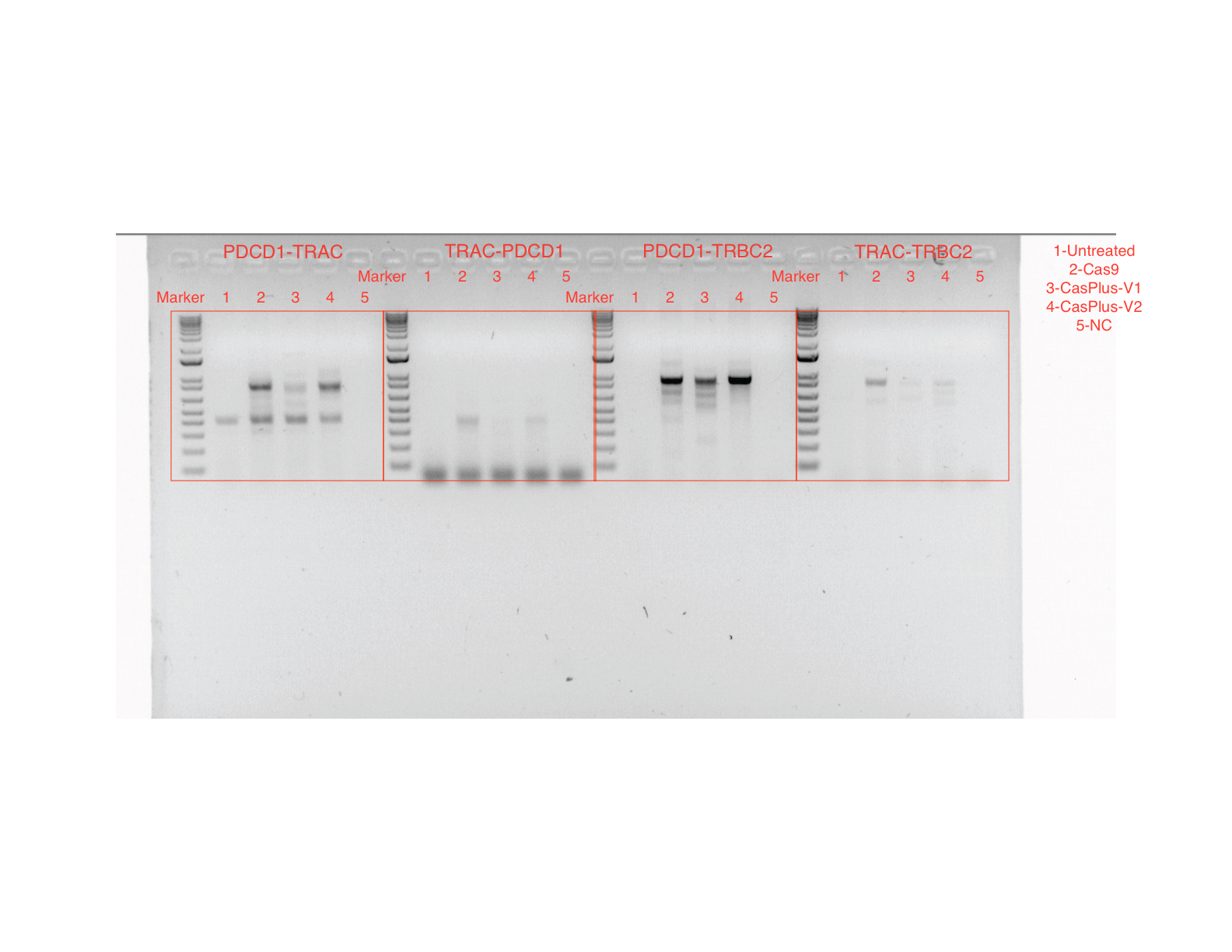

Supplement: Supplementary file 8 — Source data Fig. 6 [file 44318_2024_158_MOESM8_ESM.zip › SD figure 6/6F/6F-1.tiff]

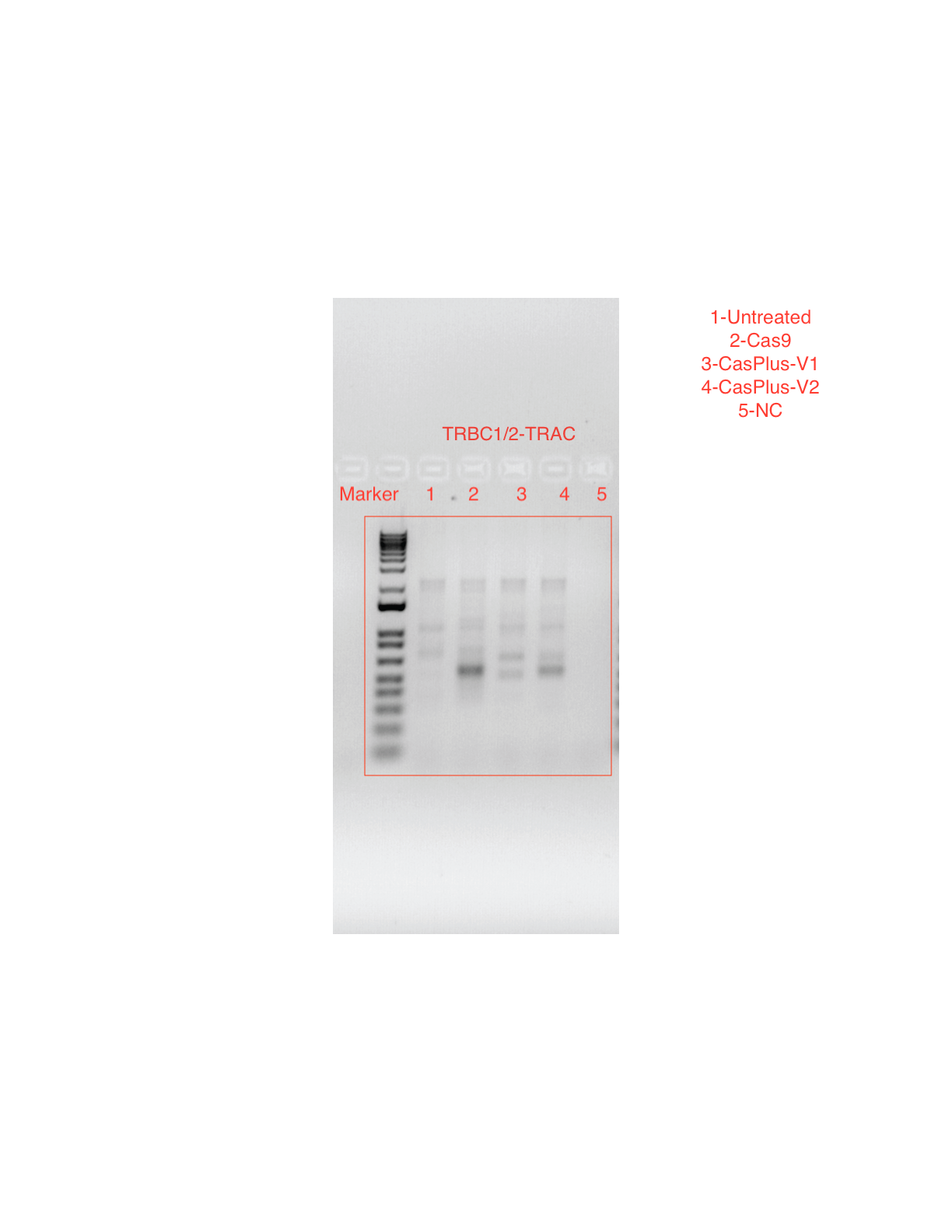

Supplement: Supplementary file 8 — Source data Fig. 6 [file 44318_2024_158_MOESM8_ESM.zip › SD figure 6/6F/6F-3.tiff]

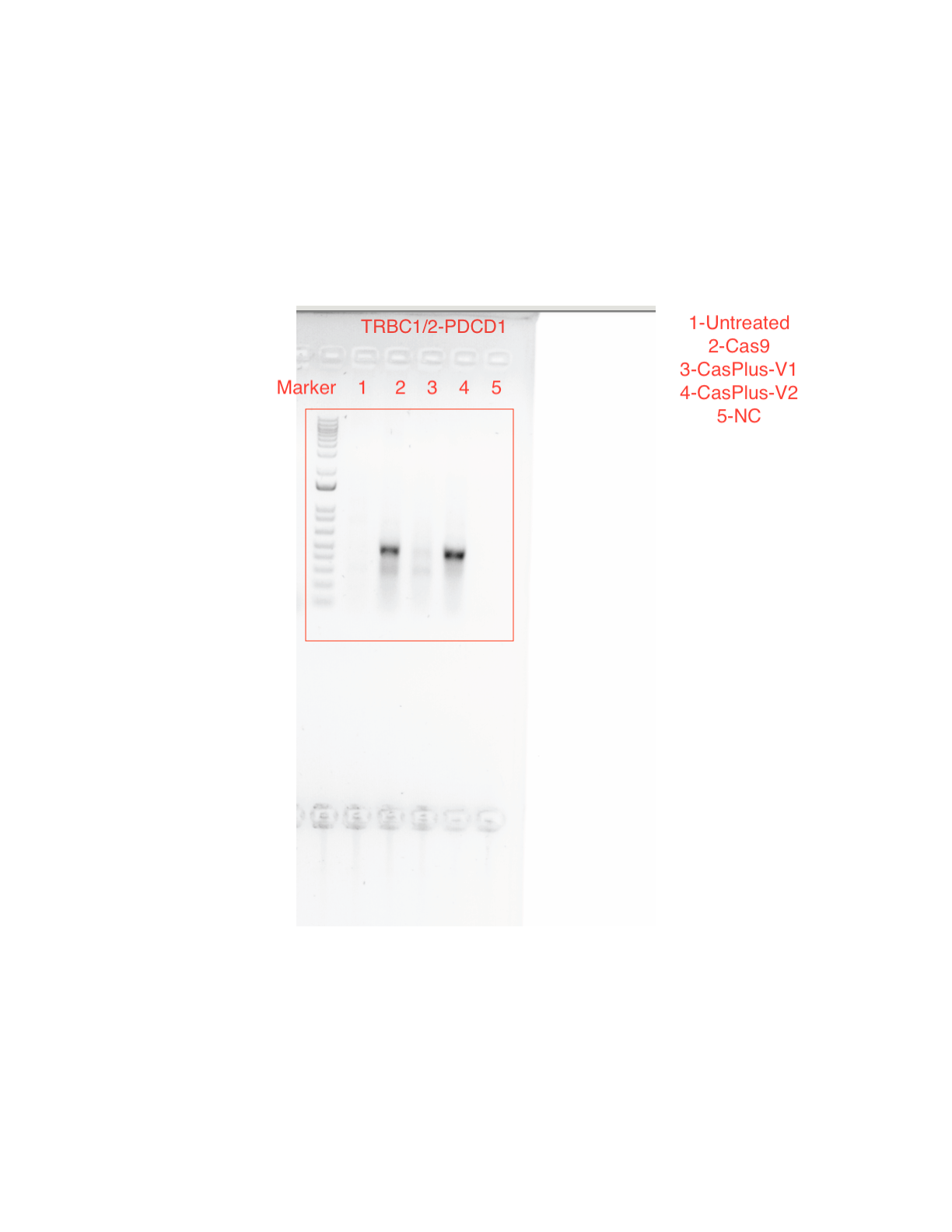

Supplement: Supplementary file 8 — Source data Fig. 6 [file 44318_2024_158_MOESM8_ESM.zip › SD figure 6/6F/6F-2.tiff]

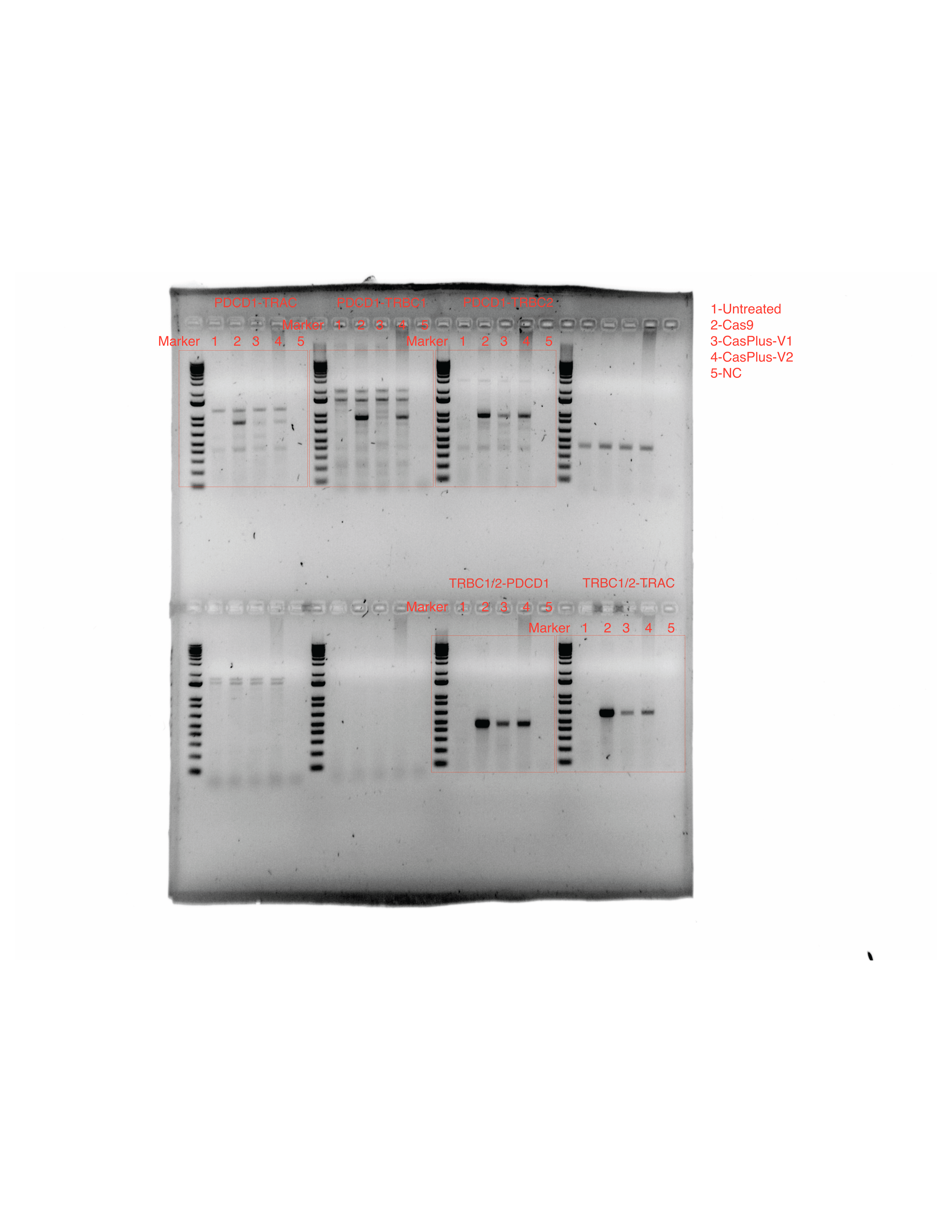

Supplement: Supplementary file 8 — Source data Fig. 6 [file 44318_2024_158_MOESM8_ESM.zip › SD figure 6/6B/6B-1.tiff]

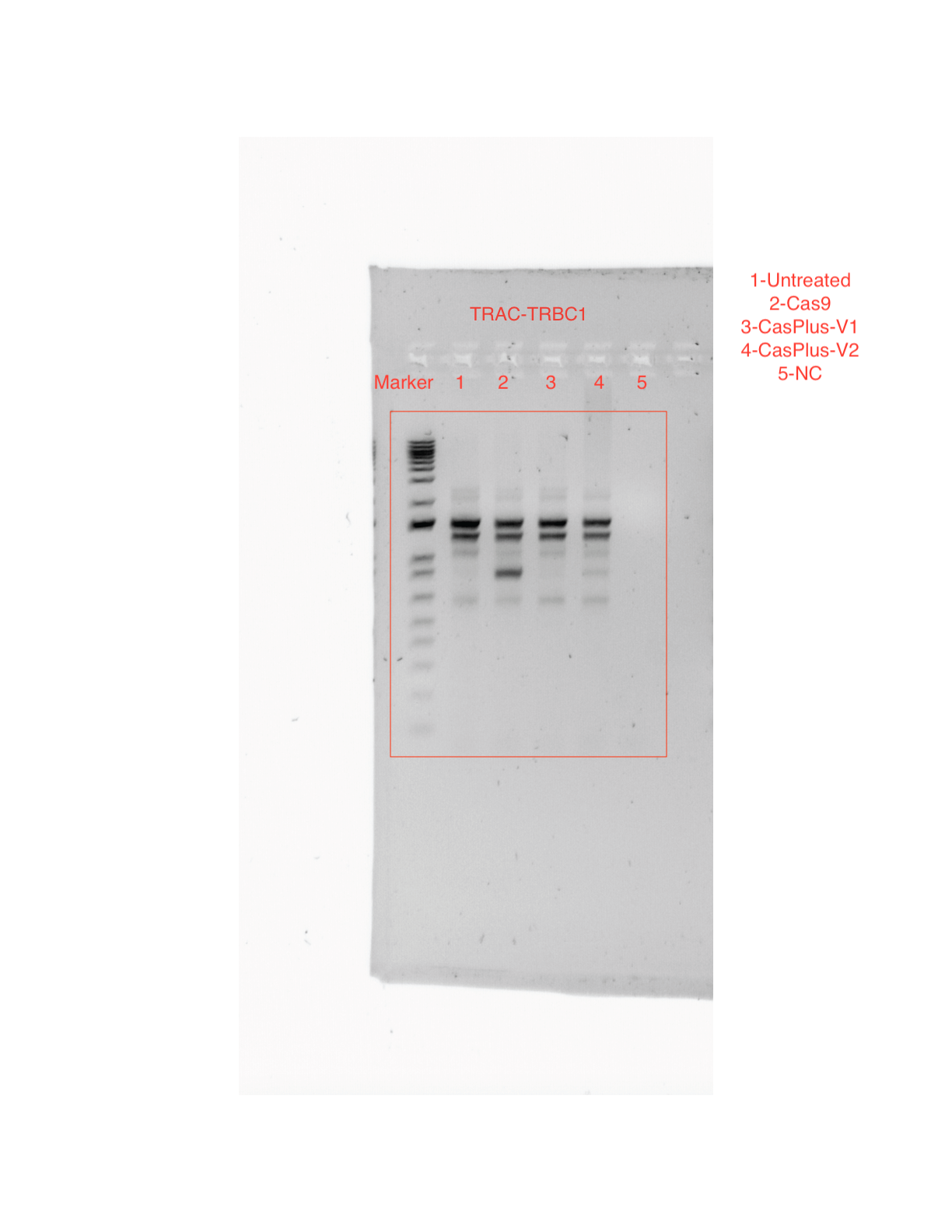

Supplement: Supplementary file 8 — Source data Fig. 6 [file 44318_2024_158_MOESM8_ESM.zip › SD figure 6/6B/6B-2.tiff]

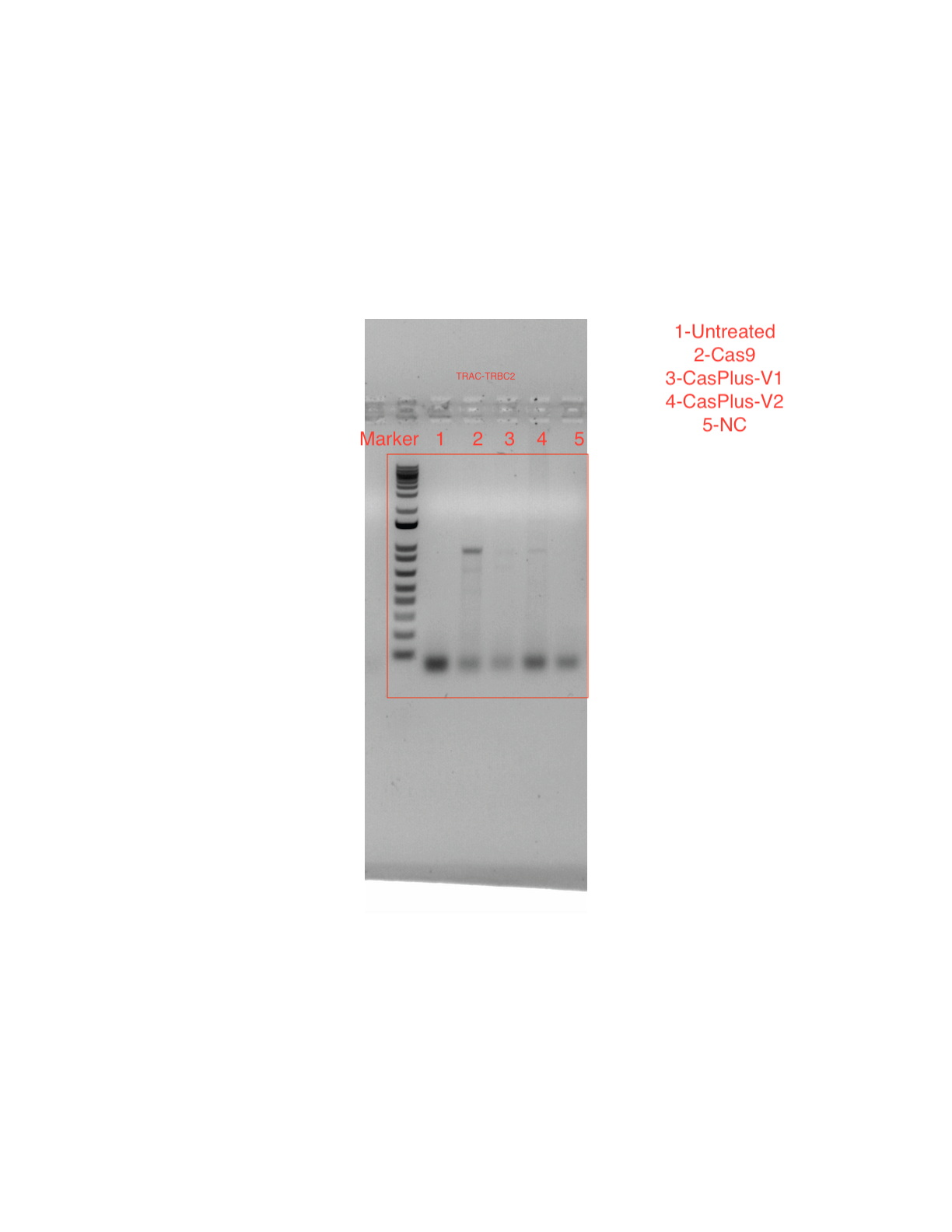

Supplement: Supplementary file 8 — Source data Fig. 6 [file 44318_2024_158_MOESM8_ESM.zip › SD figure 6/6B/6B-3.tiff]
